# Basal Cell Carcinoma With Matrical Differentiation: A Case Report and Literature Review

**DOI:** 10.1177/10668969251384328

**Published:** 2025-11-10

**Authors:** Samantha Keow, Joshua Del Papa, Matthew Cecchini, Anurag Sharma

**Affiliations:** 1 Michael G. DeGroote School of Medicine, 12362 McMaster University, Hamilton, ON, Canada; 2 Department of Pathology and Laboratory Medicine, 6221 Western University, London, ON, Canada; 3 646021 Department of Pathology and Laboratory Medicine, London Health Sciences Centre, London, ON, Canada

**Keywords:** basal cell carcinoma with matrical differentiation, BCC-MD, basal cell carcinoma, matrical differentiation, pilomatrix carcinoma, immunohistochemistry

## Abstract

Basal cell carcinoma (BCC) with matrical differentiation is an extremely rare subtype of basal cell carcinoma. We present an example of BCC with matrical differentiation and review the relevant literature. A 75-year-old man presented with a rapidly enlarging nodule on his forehead, with the clinical diagnosis of squamous cell carcinoma. Given atypical matrical proliferation on initial biopsy, there was concern for pilomatrix carcinoma. However, a repeat biopsy confirmed a diagnosis of BCC with matrical differentiation. Immunohistochemically, the BCC lobules expressed BCL2 and BER-EP4, while the areas of matrical differentiation showed nuclear β-catenin expression. The tumor cells were negative for keratin 7. BCC with matrical differentiation predominantly affects men, with a mean age of 70 years. Most tumors occur in the head and neck area, and lesions are often slow-growing or asymptomatic. Differential diagnosis includes pilomatrix carcinoma. Outcomes are generally favorable following surgical excision, although 2 instances of lymph node metastasis have been reported.

## Introduction

Basal cell carcinoma (BCC) is the most common type of skin cancer. The pathological subtype of BCC with matrical differentiation is extremely rare, with only 58 tumors reported in detail.^1–18^ BCC with matrical differentiation is a slow-growing, asymptomatic neoplasm generally associated with favorable outcomes. These tumors exhibit typical diagnostic features of BCC, as well as basaloid nests containing shadow cells indicative of hair-matrix differentiation. Such pathological characteristics are also observed in pilomatricoma, a benign neoplasm with clinical features similar to BCC with matrical differentiation.^19^ However, these features may also be present in pilomatrix carcinoma, a malignant and aggressive transformation of pilomatricoma associated with high rates of recurrence and metastasis.^20^ This highlights the critical need for accurate differentiation between these entities. We report BCC with matrical differentiation in a 75-year-old man and review the relevant literature.

## Case Report

A 75-year-old man presented with a rapidly enlarging white-tan nodule measuring 1.6 × 1.4 × 1.0 cm on his forehead. Clinically, the lesion was diagnosed as squamous cell carcinoma (SCC), and a superficial shave biopsy was performed. The biopsy revealed atypical matrical proliferation, and the lesion was diagnosed as an irritated pilomatricoma with reactive changes. However, the presence of atypical squamoid and basaloid cells raised concerns about the possibility of a rare pilomatrix carcinoma. As a result, complete excision with further comprehensive pathological evaluation was recommended.

**Figure 1.**
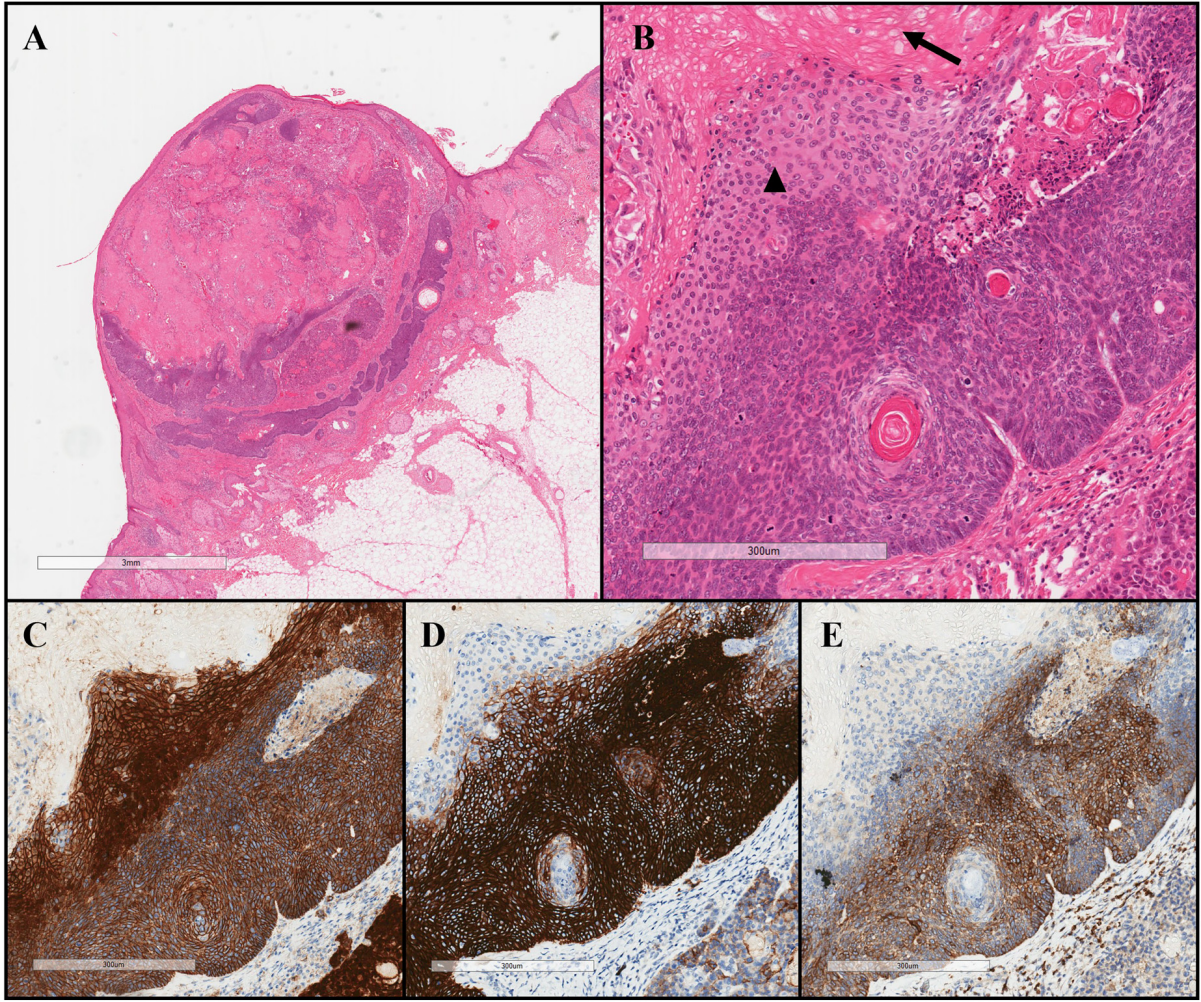
Basal cell carcinoma with matrical differentiation. (A) and (B) An exophytic subepithelial basaloid nodule with nodular basal cell carcinoma transitioning to areas of matrical differentiation. Ghost cells (arrow) and intermediary cells (arrowhead) are identified. (C) Areas of matrical differentiation show nuclear positivity to β-catenin. Areas of classical basal cell carcinoma show positive cytoplasmic BER-EP4 (D) and BCL2 (E).

The lesion was surgically excised, and further microscopic examination confirmed a diagnosis of BCC with matrical differentiation. Sections showed a well-circumscribed exophytic nodular basaloid lesion (Figure 1A). The basaloid nests, consistent with nodular basal cell carcinoma, exhibit peripheral palisading and retraction artifact and are immunoreactive for BER-EP4 and BCL2 (Figure 1D and E). The tumor transitioned into an area with matrical differentiation, characterized by more eosinophilic intermediary cells undergoing abrupt keratinization into eosinophilic ghost cells (Figure 1B). The matrical and intermediate cells demonstrate nuclear β-catenin positivity (Figure 1C). The tumor cells were negative for keratin 7 (not shown).

## Discussion

Since BCC with matrical differentiation was first described in 1988,^9^ our literature review has identified only 58 previous instances,^1–17^ including 11 from a study on matrical tumors.^18^ Among the case reports that provided patient demographics, 63% of the patients were men, with a mean age of 70 years (range: 41-94). The most common site for these tumors was the head and neck, with 48.8% occurring in this region. Specifically, 23.3% were on the forehead/scalp, 9.3% on the ear/periauricular area, 9.3% on the nose, 2.3% on the cheek, and 4.7% on unspecified areas on the head or neck. Other areas of development included the torso (20.9%), upper limb (16.3%), lower limb (9.3%), and axilla/upper chest (4.7%).

BCCs with matrical differentiation were frequently characterized as nodular and erythematous to flesh-colored, with a pearly or ulcerative appearance. Lesions varied in size from 5 to 110 mm, with an average measurement of 23.9 mm. Clinical diagnoses were documented in 12 patients, most commonly reporting BCC,^2,4,7,9,11,14^ and less frequently SCC,^5,7^ keratoacanthoma,^5^ epithelioma,^9^ actinic keratosis, or psoriasis vulgaris.^3^ Before excision, the lesions were present from 2 months^7,10^ to 20 years,^2^ and were often slow-growing or asymptomatic. Seven patients had a previous history of skin cancer or precancerous skin lesions, including BCCs,^2,5,6,8,10^ SCCs,^6,10^ and actinic keratoses.^2^ Two patients were chronically immunosuppressed following an orthotopic heart transplant 10 years earlier and a renal transplant 7 years earlier, respectively.

Histologically, the proportion of BCC and matrical components varies significantly among BCCs with matrical differentiation.^7^ Features characteristic of classical BCC were observed, including basaloid tumor islands with follicular germinative cells and tumor cells displaying narrow or scant cytoplasm, uniform nuclei that were oval to elongated, and peripheral palisading. These islands were frequently separated from the stroma by retraction clefts and were either connected to the epidermis or extended into the reticular dermis and subcutaneous fat. In 5 tumors, cystic changes within larger BCC lobules were noted,^1,5,7,11,17^ with some clear spaces containing acantholytic and dyskeratotic cells, and concentric keratin laminations. Several features of follicular matrix differentiation were also noted, such as cystic cavities within BCC lobules containing central pale aggregates of shadow cells with distinct borders and central unstained areas. There was often an abrupt transition from basaloid cells to shadow cells. Eosinophilic trichohyaline granules were observed in 20 tumors.^4,5,7^ Intratumoral melanin was noted in 3 tumors,^7,16,17^ and calcium deposits within shadow cells were noted in 7.^5,7^ The surrounding stroma commonly exhibited chronic inflammation, congested capillaries, and mild desmoplasia. Although mitoses were generally rare, one tumor showed frequent mitoses associated with severe anaplasia and dyskeratotic cells.^17^

Immunohistochemically, BCC lobules frequently expressed BER-EP4, BCL2, keratin, and weak or focal membranous β-catenin, while being negative for PHLDA1, CD34, EMA, K15, and K20.^6,7,11^ Areas of matrical differentiation often showed strong nuclear or cytoplasmic β-catenin, especially accentuated at the palisaded edge of tumor nodules.^6,7,11,14^ These areas also demonstrated focal PHLDA-1 in cells surrounding shadow cells,^11,18^ along with focal BER-EP4^11^ and EMA,^7^ weak BCL2,^11^ CD10 (MME) in the epithelium,^7,11^ and varied but generally stronger Ki-67/MIB-1 expression compared to BCC areas.^11^ Focal cytoplasmic osteopontin staining has been reported in the basaloid cells of 2 tumors.^8^ Hair keratins 35 and 31 have been observed in transitional cells and lower shadow cells, and LEF1 has been found in the nuclei of germinative matrix cells.^18^

Five tumors reported mutations in the *CTNNB1* gene, including 4 instances with *S37F* mutations in exon 3^7,13^ and 1 with a deletion in exon 3.^15^ One reported a 550 C > T *PTCH1* mutation that resulted in a premature stop codon.^7^ Previous genetic studies have also identified mutations in *KIT*, *TP53*, *SMAD4*, and *ERBB4* associated with BCC with matrical differentiation.^7^

When atypia is observed in the matrical component, pilomatrix carcinoma must be considered in the differential diagnosis. Pilomatrix carcinoma displays several malignant features absent in the matrical component of BCC with matrical differentiation, such as high mitotic activity with atypical mitotic figures, prominent necrosis, and an infiltrative growth pattern. Furthermore, pilomatrix carcinoma lacks a typical BCC component.^21^

The definitive treatment for BCC with matrical differentiation is surgical excision. Eight documented tumors reported no recurrence 1 to 6 years after surgical excision,^2,8,9,14^ while an additional 2 showed no recurrence 6 to 8 years after surgical excision with local radiotherapy.^2^ One tumor, excised using the Mohs procedure, had no evidence of residual disease after 18 months.^6^ Although outcomes are generally favorable, 2 patients developed lymph node metastases: 1 nasal tumor metastasized to the left submandibular hypermetabolic lymph node after multiple local excisions and recurrences,^14^ and the another involved a large axillary fungating tumor with deep subcutaneous extension and lymphovascular invasion.^7^

We present a rare instance of BCC with matrical differentiation, clinically diagnosed as SCC, in a 75-year-old man. Our literature review indicates that BCC with matrical differentiation predominantly affects men, with a mean age of 70 years, and occurring in the head and neck region. Although BCC with matrical differentiation is typically slow-growing with generally favorable outcomes, its histological similarities to pilomatrix carcinoma—a far more aggressive and deadly hair matrix neoplasm—underscores the importance of accurate differentiation. This tumor adds to the limited literature on BCC with matrical differentiation by providing valuable clinical, pathological, and immunohistochemical insights to aid in its recognition and diagnosis.
